# Toward Mechanistic Design of Surrogate Buffers for Dissolution Testing of pH-Dependent Drug Delivery Systems

**DOI:** 10.3390/pharmaceutics12121197

**Published:** 2020-12-10

**Authors:** Johannes Andreas Blechar, Jozef Al-Gousous, Christoph Wilhelmy, Annika Marielina Postina, Marcus Getto, Peter Langguth

**Affiliations:** 1 Department of Biopharmaceutics and Pharmaceutical Technology, Johannes Gutenberg University Mainz, 55099 Mainz, Germany; jblechar@uni-mainz.de (J.A.B.); joalgous@uni-mainz.de (J.A.-G.); christophwilhelmy@web.de (C.W.); 2 Department of Pharmaceutical Sciences, University of Michigan, Ann Arbor, MI 48104, USA; 3 Institute of Pharmaceutical and Biomedical Sciences, Johannes Gutenberg University Mainz, 55099 Mainz, Germany; apostina@students.uni-mainz.de (A.M.P.); marcus.getto@students.uni-mainz.de (M.G.)

**Keywords:** enteric coating, dissolution, bicarbonate, succinate, citrate, surrogate buffer, biorelevant, Eudragit, HPMCP, HPMCAS

## Abstract

The in vivo dissolution of enteric-coated (EC) products is often overestimated by compendial in vitro dissolution experiments. It is of great interest to mimic the in vivo conditions as closely as possible in vitro in order to predict the in vivo behavior of EC dosage forms. The reason behind this is the overly high buffering capacity of the common compendial buffers compared to the intestinal bicarbonate buffer. However, a bicarbonate-based buffer is technically difficult to handle due to the need for continuous sparging of the media with CO_2_ to maintain the desired buffer pH. Therefore, bicarbonate buffers are not commonly used in routine practice and a non-volatile alternative is of interest. A mathematical mass transport modelling approach was previously found to enable accurate calculation of surrogate buffer molarities for small molecule compounds; however, the additional complexity of polymeric materials makes this difficult to achieve for an enteric coat. In this work, an approach was developed allowing relatively rapid screening of potential surrogate buffers for enteric coating. It was found that the effective buffering pKa of bicarbonate at the surface of a dissolving enteric polymer tended to be around 5.5, becoming higher when the dissolving enteric polymer formed a gel of greater firmness/viscosity and vice versa. Using succinate (pKa 5.2 under physiological ionic strength) and/or citrate (pKa 5.7 under physiological ionic strength) at conjugate base molarities corresponding to bicarbonate molarities in the intestinal segments of interest as an initial “guess” can minimize the number of experimental iterations necessary to design an appropriate surrogate.

## 1. Introduction

An enteric coating is usually based on a polycarboxylic film former that protects the active pharmaceutical ingredient (API) from degradation in the gastric fluid or reduces the risk of adverse side effects by delaying the drug release until after gastric emptying [1]. In the fasted gastric environment, the enteric polymer is unionized, thus insoluble, and prevents the dosage form from disintegration and release of the API. After being emptied from the stomach, the enteric-coated (EC) dosage form is exposed to intestinal fluid with increased pH that is mostly buffered by bicarbonate [2,3,4]. When the surface pH (pH_0_) of the EC dosage form is higher than the dissolution pH threshold of the polymer (which is dependent on the polymer’s pKa), the acidic groups in the enteric coating become deprotonated, promoting disentanglement and dissolution of the polymer and ultimately leading to the disintegration and dissolution of the dosage form.

Although being known for over a century, enteric coatings in pharmaceutical dosage forms often suffer from poor in vivo performance [1,5]. One reason for the deficient in vivo performance is the use of dissolution buffers for in vitro testing with poor biopredictivity, which precludes proper in vitro product evaluation [5]. Compendial dissolution media for testing of EC dosage forms usually consists of relatively concentrated phosphate buffers [6] compared to intestinal bicarbonate concentrations [7]. Consequently, the buffering activity of the pharmacopoeian buffers is much higher, promoting a faster dissolution in vitro than in vivo. This is because, in contrast to its high buffer capacity in bulk [8], bicarbonate exhibits weak buffering activity at the surface of the dissolving ionizable solid [9]. The reason behind that is the relatively slow kinetics of the hydration and dehydration reactions of CO_2_ and H_2_CO_3_, respectively, as depicted in Equation (1):

(1) $${\mathrm{CO}}_{2}\left(g\right)⇌{\mathrm{CO}}_{2}\left(\mathrm{aq}\right)\begin{array}{c} \mathrm{Hydration}\\ ⇌\\ \mathrm{Dehydration} \end{array}H_{2}{\mathrm{CO}}_{3}\left(\mathrm{aq}\right)⇌H^{+}\left(\mathrm{aq}\right)+{{\mathrm{HCO}}_{3}}^{-}\left(\mathrm{aq}\right)$$

The intrinsic pKa of the H_2_CO_3_ ionization is around 3.3 [9], while a potentiometric determination will result in an apparent pKa of 6.04 because of the H_2_CO_3_ being in an additional equilibrium with CO_2_ [10]. Compared to the hydration and dehydration of CO_2_ and H_2_CO_3_, the titration process is too slow to disrupt this equilibrium in the bulk solution. Therefore, CO_2_ acts as if it were the conjugate acid for HCO_3_^−^, with H_2_CO_3_ appearing to be confined to the role of a short-lived intermediate. Accordingly, the apparent Ka will be a product of the intrinsic Ka and the hydration equilibrium constant (equal to the quotient of the first order rate constants of the hydration and dehydration reactions, respectively) resulting in this pKa of 6.04 [9].

However, in the diffusion boundary layer around a dissolving ionizable solid, the diffusional times of CO_2_ and H_2_CO_3_ are too short for the hydration and dehydration reactions governing the interconversion of these two species to remain at equilibrium [9]. Actually, the dehydration reaction is only marginally faster than ordinary diffusional processes (around 50 times compared to million times for H_2_CO_3_ deprotonation), while the hydration reaction is even slower. As a result, the effective apparent pKa at which bicarbonate will buffer the surface of a dissolving ionizable solid (and thus govern the dissolution rate) will take an intermediate value between the mere carbonic acid ionization value of 3.3 and the bulk equilibrium value of 6.04. This will push this effective interfacial buffering pKa too much below the pH of intestinal fluid for bicarbonate to strongly buffer the dissolving solid surface. As a result of this weak buffering action, the gap between bulk and surface pH values will be relatively large, thus impeding prompt dissolution.

However, owing to the volatility of dissolved CO_2_, bicarbonate buffer requires continuous sparging and pH monitoring, making its routine use technically difficult and even impossible in some devices such as disintegration and reciprocal cylinder apparatuses. Therefore, developing a non-volatile surrogate buffer, the performance of which is comparable to bicarbonate, is desirable. In this regard, Hofmann et al. [11] successfully developed a phosphate-based surrogate for the dissolution of ibuprofen suspensions by calculating an equivalent phosphate molarity using the reversible non-equilibrium (RNE) model developed by Al-Gousous et al. [9].

Since enteric polymers entail a much greater degree of complexity than small molecules such as ibuprofen (due to polymer relaxation and swelling as well as the lack of a well-defined pKa owing to each ionization weakening the next one), such direct calculation is not possible. However, Al-Gousous et al. managed through semi-empirical experimental matching of phosphate and bicarbonate to develop a phosphate-based surrogate buffer for enteric-coated tablets that predicted the in vivo behavior of enteric-coated aspirin tablets fairly well [12]. However, a more mechanistic approach will enable easier surrogate buffer development for a wide range of coatings targeting different intestinal segments with minimal experimental iterations.

In this regard, a starting point is provided by Equation (2) [9]:

(2) $${\mathrm{pKa}}_{\mathrm{eff}}=3.3+\log\left(1+\frac{D_{{\mathrm{CO}}_{2}}}{D_{H_{2}{\mathrm{CO}}_{3}}}\times \frac{k_{d}}{k_{h}+\frac{2D_{{\mathrm{CO}}_{2}}}{h^{2}}}\right)$$

where the D terms are the diffusivities of the species in the subscripts; h is the boundary layer thickness; and k_d_ and k_h_ are the first order rate constants for the H_2_CO_3_ dehydration and CO_2_ hydration reactions, respectively. This equation gives pKa_eff_, the apparent effective buffering pKa in the boundary diffusion layer around a dissolving ionizable solid. For small molecule compounds under regular hydrodynamic conditions [13], this pKa_eff_ of bicarbonate will lie between 4 and 5. While, as explained later in this manuscript, the more complicated behavior of polymers compared to small molecule compounds makes it difficult to make a direct calculation, this estimate serves as a good starting point in the quest toward a systematic approach to design appropriate surrogate buffers, which is the aim of this work.

## 2. Materials and Methods

### 2.1. Materials

Delayed release mesalamine 500 mg tablets (Claversal, Recordati Pharma GmbH, Ulm, Germany) and diclofenac sodium 50 mg tablets (Diclofenac AbZ, AbZ-Pharma GmbH, Ulm, Germany) were brought from a local pharmacy. Diclofenac sodium was obtained from Cayman Chemical Company (Ann Arbor, MI, USA), mesalamine was obtained from Sigma-Aldrich Chemie GmbH (Steinheim, Germany), and paracetamol was obtained from Caesar and Loretz GmbH (Hilden, Germany). Transparent size 0 hydroxypropyl methylcellulose (HPMC) hard shell capsules (ACG Nature Caps Plus) were gifted from ACG Associated Capsules Pvt Ltd. (Ashagadh, Maharashtra, India). Methacrylic acid and methyl methacrylate copolymer (1:2) NF (Eudragit S) was received as a free sample from Evonik Röhm GmbH (Darmstadt, Germany). Hypromellose acetate succinate (HPMCAS-LF) and hypromellose phthalate (HP-50, HP-55, and HP-55S) were received as free samples from Shin-Etsu Co., Ltd. (Tokyo, Japan). Triethyl citrate (TEC) was obtained from Merck KGaA (Darmstadt, Germany) and talc was purchased from Euro OTC Pharma GmbH (Bönen, Germany). All other substances were of analytical grade.

### 2.2. Manufacturing of EC Hard Shell Capsules

Paracetamol was filled manually into the HPMC hard shell capsules (aponorm Kapselfüllgerät, WEPA Apothekenbedarf GmbH and CO. KG, Hillscheid, Germany). All capsule batches passed the mass uniformity test (2.9.5) according to the European pharmacopoeia (Ph. Eur. 9.0). Table 1 summarizes the average dose of each batch.

The paracetamol-filled capsules were coated in a Labotech Capsule Coater (Labotech, The Netherlands) [14]. For the preparation of the coating dispersions, we dispersed 1.152 g TEC, 2.88 g talc, and 5.76 g of the enteric polymer in 240 g acetone.

Coating was performed at the following parameters: 30 capsules per batch, air pressure of 2.0 bar, input of the coating dispersion of 0.8–0.9 g/min, and length of dipping tube 6.6 cm. All coatings were performed at room temperature. Following the application of a predefined amount of coating liquid, we continued the process for 10 further minutes without input of the coating dispersion. At the end, the capsules were placed on aluminum foil and dried overnight at room temperature. Coating levels based on the dry polymer weight are summarized in Table 1.

The preparation of the caffeine-filled HPMC capsules was described in a previous publication [15]. Each capsule had a dose of 73.5 ± 4.0 mg of caffeine and passed the content uniformity test (2.9.6.) according to the European pharmacopoeia (Ph. Eur. 9.0). The coating level was 10 mg/cm^2^ (based on dry polymer mass).

### 2.3. Dissolution Test

Dissolution tests were performed using the PTW S III dissolution tester (PharmaTest, Hainburg, Germany). Capsules were tested in a basket apparatus (United States Pharmacopoeia (USP) apparatus I) at 100 rpm and tablets in a paddle apparatus (USP apparatus II) at 50 rpm (unless otherwise stated). A total of 900 mL of dissolution media was used at 37 °C. At predefined times, 5 mL samples were withdrawn. The samples were filtered through 0.45 µm PTFE filters (obtained from Carl Roth GmbH, Karlsruhe, Germany). The sample volume was replaced with blank buffer. Samples were quantified spectrophotometrically (UV–visible spectrophotometer, UV-6300PC, VWR International) at drug-specific wavelengths (diclofenac, 276 nm; mesalamine, 311 nm; paracetamol, 243 nm; and caffeine, 275 nm). During the experiment with bicarbonate buffer, the dissolution media was sparged with a mixture of CO_2_ and air to adjust the pH accordingly. The ionic strength of all buffers was adjusted to 154 mM with NaCl. All experiments were performed in triplicate, if not stated otherwise. The time required to achieve 5% release (t_5%_) was calculated by linear interpolation and used for comparison. This parameter is most representative of the coat dissolution rather than the whole dissolution profile [16]. Data are given as mean value ± standard deviation (SD).

### 2.4. Buffer Preparation

Dissolution media were prepared at room temperature. Deionized water was deaerated using a PT-DDS 4 (PharmaTest, Hainburg, Germany) at 30 °C for 2 h. The water was allowed to cool down overnight. After dissolving the buffer salts in degassed water, we adjusted the pH to the desired value (±0.05) using 0.1 N HCl/NaOH. The pH values were also checked after reaching a temperature of 37 °C. The pH of bicarbonate buffers was adjusted with a mixture of CO_2_ and air in the dissolution apparatus, as described by Al-Gousous et al [16].

### 2.5. Statistical Analysis

Statistical evaluation was performed using IBM SPSS statistics version 23 (IBM Corporation, Armonk, NY, USA). *p*-value < 0.05 was assumed to be significant.

## 3. Results and Discussion

### 3.1. Dissolution of Mesalamine Tablets Coated with a Mixture of Methacrylic Acid/Methyl Methacrylate Copolymer (1:2) and Methacrylic Acid/Methyl Methacrylate Copolymer (1:1)

**Figure 1:**
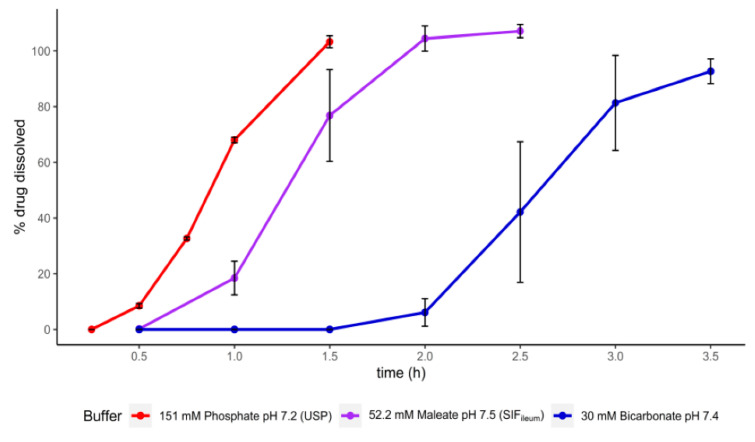
Dissolution of Claversal tablets in different buffers representing the distal intestine.

The coating of Claversal tablets is composed of methacrylic acid/methyl methacrylate copolymer (1:2) and methacrylic acid/methyl methacrylate copolymer (1:1) and targets the drug release in the distal intestine. Claversal tablets were first tested in USP buffer (151 mM phosphate buffer (pH 7.2)) and the simulated intestinal fluid (SIF_ileum)_ media (52.8 mM maleate buffer (pH 7.5)) [17], which are both intended to simulate the distal intestine. To compare the release in the simulated media to the in vivo situation, we also performed dissolution experiments in an ileal fluid-based bicarbonate buffer (30 mM (pH 7.4)) [18]. Both buffers—the USP phosphate buffer and the SIF_ileum_—strongly overestimated the onset of mesalamine release from the tablets compared to the release in bicarbonate buffer (Figure 1).

The dissolution of a carboxylic acid is known to be dependent on the pH and the buffering capacity of the medium. This buffer capacity depends on the molarity of the buffer and its pKa. With increasing buffer molarity, the dissolution is generally accelerated over a broad range of buffer molarity [19]. Additionally, the discrepancy between the dissolution rates of the API in the three buffers is due to the different pKa values of the buffer species within the diffusion layer. The pKa of phosphate and maleate at physiological ionic strength are 6.8 [20] and 5.8 (intrinsic pKa 6.22 [21] corrected for the ionic strength using the Debye–Hückel theory [22]), respectively, whereas the pKa_eff_ of bicarbonate is expected at 4 to 5 at typical hydrodynamic conditions [23]. Therefore, the dissolution of enteric coatings in the intestinal fluid that is buffered by bicarbonate is often slower than the tested in vitro dissolution in compendial media.

In the case of EC products, the slower dissolution associated with decreased buffer capacity usually results in an increase in the discriminatory power of the test since differences between individual dosing units become more pronounced. This is supported by EC product dissolution data from this study as well as other ones [12,24], showing larger error bars in lower capacity buffers.

These results are in line with the findings of Al-Gousous et al. [16], where the dissolution of Delzicol tablets was tested in bicarbonate, phosphate, and maleate buffer at similar conjugate base concentrations and comparable pH values. The fastest dissolution was found in the phosphate-based media followed by the maleate buffer. The slowest dissolution rate was found in the bicarbonate buffer. The main difference in the dissolution media were the pKa values of the buffers tested. As explained by Al-Gousous et al., a maleate buffer is more effective maintaining the surface pH of a dissolving enteric coating at a near neutral value than a bicarbonate buffer [16].

**Figure 2:**
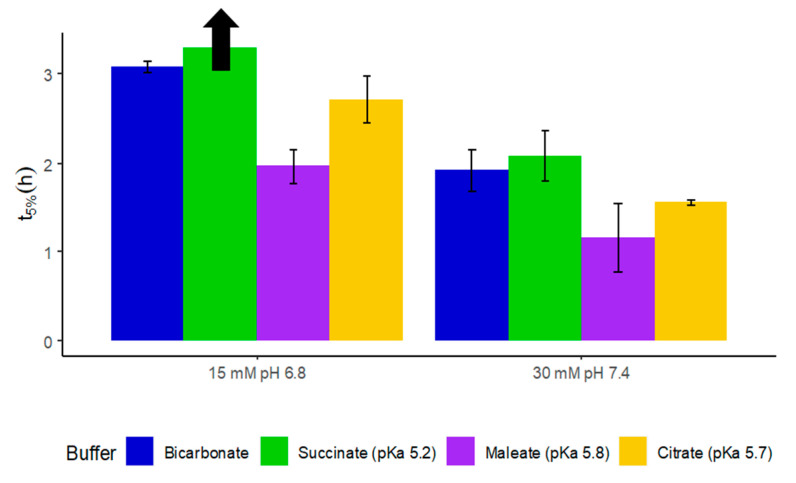
t_5%_ for Claversal tablets in different buffers at 15 mM conjugate base and pH 6.8 and at 30 mM conjugate base and pH 7.4. Dissolution in 15 mM succinate buffer took > 4 h to detect first signs of drug release (indicated by the black arrow).

To find a buffer species that promotes the dissolution similar to bicarbonate, further dissolution experiments were performed in maleate (pKa 5.8), citrate (pKa 5.7), succinate (pKa 5.2), and acetate (pKa 4.6) buffers at 30 mM and 15 mM conjugate base and pH 7.4. Even at a lower buffer molarity compared to the SIF_ileum_, the 30 mM maleate buffer overestimated the release rate (t_5%_) of mesalamine (Figure 2) compared to the ileal fluid-based bicarbonate buffer. The difference between t_5%_ (bicarbonate) and t_5%_ (maleate) was approximately 40% (difference of 45 min, *p*-value 0.025). The 30 mM citrate buffer simulated the dissolution behavior of Claversal in bicarbonate better than the maleate buffer, although the difference until 5% of the dose was released was still 18.6% (21 min, *p*-value 0.437). The 30 mM succinate buffer showed only an 8% (9 min, *p*-value 0.921) difference in the t_5%_ to bicarbonate. The acetate buffer exhibited poor bulk pH control, and first signs of coat rupture were not observed even after >3 h of testing.

**Figure 3:**
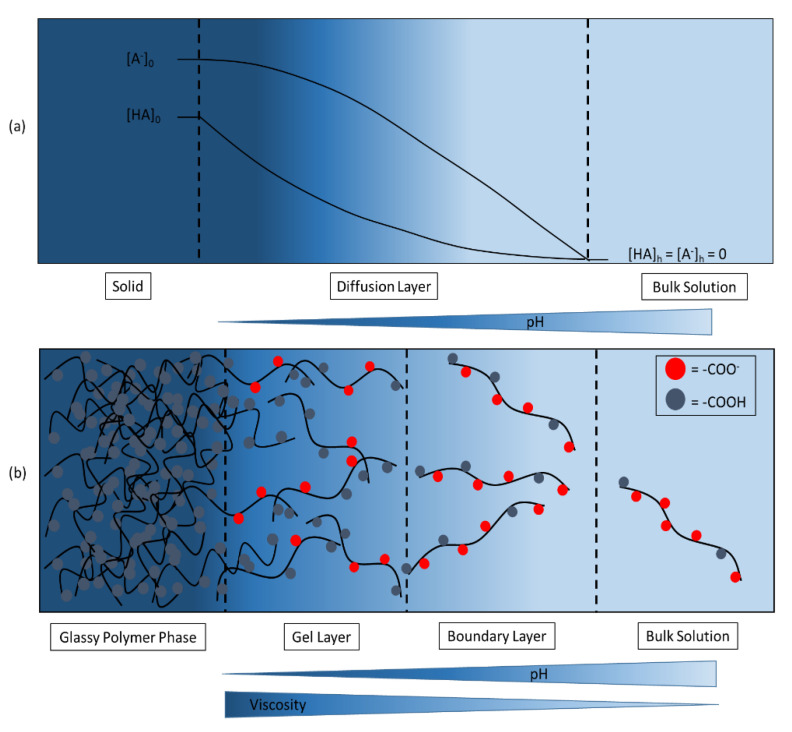
Schematic representation of (**a**) the dissolution of a solid small molecular carboxylic acid (HA) into a buffered dissolution media, adapted with permission from [19], Elsevier, 1981 and (**b**) the dissolution of an enteric polymer into a buffered dissolution media, adapted with permission from [25], AlChE, 2005.

As mentioned previously, for the dissolution of small molecules, the pKa_eff_ of bicarbonate in the diffusion layer is around 4 to 5. Knowing this, acetate buffer should promote the release of an API from an enteric-coated dosage form not much differently from bicarbonate buffer. However, different to small molecules where there is only a diffusion layer around a dissolving particle (Figure 3a), a viscoelastic gel layer is formed on a polymer’s surface (Figure 3b) into which the buffer can diffuse (in contrast to the negligible diffusion into a crystalline solid in the case of a small molecule). Within the gel layer, molecules are exposed to an increased diffusional resistance that reduces the diffusion rate and consequently increases the time available for the interconversion between CO_2_ and H_2_CO_3_ to approach equilibrium. Accordingly, the effective pKa of bicarbonate in the gel layer will differ from the pKa_eff_ in the boundary layer. Ultimately, the pH_0_ will be controlled by the pKa_eff_ and the higher effective pKa in the gel layer. Therefore, we can speak of the gel layer increasing the effective interfacial buffering pKa of bicarbonate.

At 15 mM conjugate base, the t_5%_ values were expected to increase compared to the dissolution in 30 mM dissolution media. Surprisingly, the lag time for mesalamine release in succinate buffer increased drastically and it took >4 h to detect any sign of coat rupture and drug release whilst 5% of drug release was achieved at approximately 3 h in bicarbonate buffer (Figure 2). On the other hand, the 15 mM citrate buffer matched the bicarbonate conditions much better, and the difference of the initial release was only 11.8% (Figure 2; 22 min, *p*-value 0.198) while the maleate buffer overestimated the beginning of drug release (compared to in vitro bicarbonate buffer) by over 1 h (*p*-value 0.001). This was most probably because the lower interfacial pH resulted in less ionized polymer, which in turn reduced the repulsion between polymer chains and, accordingly, their extent of disentanglement. Therefore, a firmer viscoelastic gel will develop in comparison to that in the 30 mM buffer, resulting in slower diffusion; a CO_2_-H_2_CO_3_ interconversion closer to equilibrium; and, accordingly, higher interfacial effective buffering pKa closer to that of the citrate rather than the succinate. This contrast between small molecules and polymers is further emphasized when a comparison is made to the ibuprofen dissolution data of Hofmann et al. [11]. In that study, acetate at the same conjugate base molarity promoted faster ibuprofen dissolution than bicarbonate, indicating a much lower interfacial buffering pKa of bicarbonate there.

### 3.2. Dissolution of Paracetamol Capsules Coated with Eudragit S, HP-55, and HP-55S

**Figure 4:**
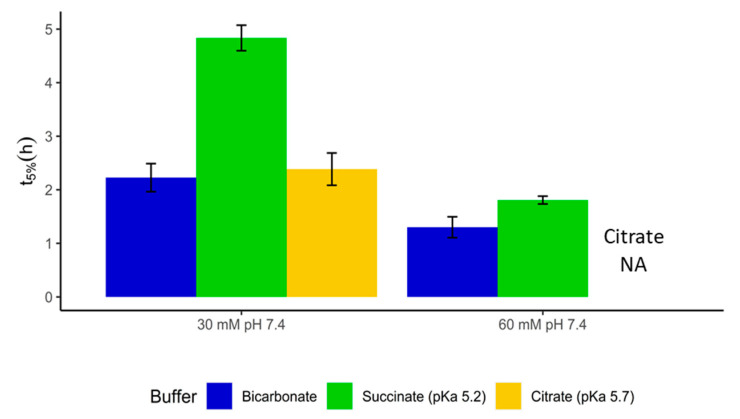
t_5%_ for paracetamol-filled capsules coated with Eudragi S in different buffers at pH 7.4. Buffer molarity is based on conjugate base. Release data in 60 mM citrate buffer are not available (NA).

Coated only with methacrylic acid/methyl methacrylate copolymer (1:2), the release of paracetamol from the capsules is expected to occur in a more distal part of the intestine, which is why 30 mM buffer (based on the conjugate base) at pH 7.4 was initially chosen for testing. A citrate-based buffer promoted the initial drug release similar to the bicarbonate buffer with only 7% (10 min, *p*-value 0.759) of difference in the initial release time; succinate, on the other hand, gave a much higher t_5%_ (Figure 4). This was most probably because here Eudragit S was not combined with the faster dissolving methacrylic acid/ethyl acrylate (1:1) copolymer, as was the case with Claversal. As a result, a firmer gel layer and a higher effective interfacial pKa of bicarbonate arose. Testing the capsules at 60 mM conjugate base molarity in both bicarbonate and succinate gave credence to this hypothesis, since the t_5%_ values were pushed much closer to each other. This can be explained as follows. At a high buffer molarity, there are more ionized groups in a polymer chain and, consequently, the electrostatic repulsion and disentanglement is increased and finally, the diffusional resistance of the gel layer is reduced. Therefore, the interfacial pKa of bicarbonate at high buffer molarity is closer to the pKa of succinate than at low molarity.

**Figure 5:**
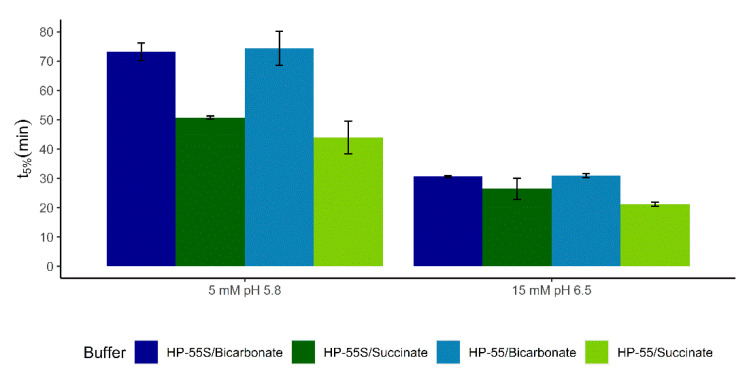
t_5%_ for paracetamol-filled capsules coated with HP-55 and HP-55S in bicarbonate and succinate buffer. Molarities are based on conjugate base.

To further test the hypothesis of the gel layer viscosity/firmness influencing the pKa_eff_ of bicarbonate, we coated capsules with HP-55 and HP-55S. HP-55 and HP-55S are chemically identical and differ only in their polymerization grade. The increased molecular weight of HP-55S should lead to a more viscous gel layer [26] and therefore increase the diffusional resistance. The decreased diffusion rate raises the pKa_eff_ of bicarbonate within the gel layer. Consequently, the difference of the initial release in bicarbonate and succinate buffer from HP-55S-coated capsules should be less than from capsules coated with HP-55. As depicted in Figure 5, while both HPMCP grades gave t_5%_ values lower than those in bicarbonate, the difference was larger for the lower molecular weight HP-55 compared to HP-55S. The statistical significance of this effect was confirmed by ANOVA testing where a significant buffer by polymer interaction was found (*p*-value 0.023). This means that bicarbonate exhibited higher interfacial pKa with the higher molecular weight HPMCP-55S, which supports our hypothesis.

### 3.3. Dissolution of Paracetamol Capsules Coated with HPMCAS-LF and Caffeine Capsules Coated with HP-50

**Figure 6:**
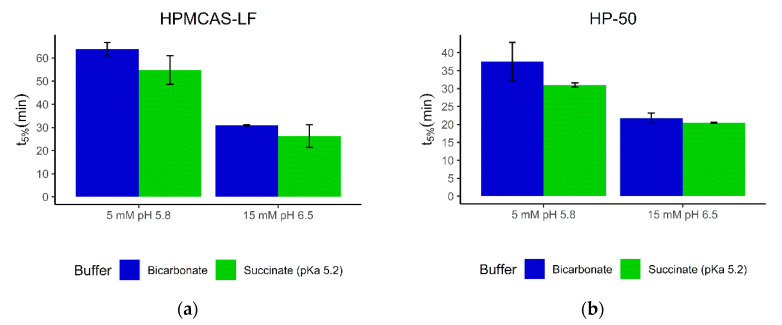
t_5%_ for (**a**) paracetamol-filled capsules coated with HPMCAS-LF in bicarbonate and succinate buffer and (**b**) caffeine-filled capsules coated with HP-50 in bicarbonate and succinate buffer. Molarities are based on conjugate base.

The two tested enteric polymers HP-50 and HPMCAS-LF had dissolution pH thresholds at 5.0 and 5.5, respectively, and the drug release was in the proximal small intestine. Accordingly, the buffers tested had 5 mM and 15 mM conjugate base and pH values of 5.8 and 6.5, respectively. As expected, the onset of drug dissolution in 15 mM buffer was significantly earlier than in 5 mM buffer. However, the differences between the bicarbonate and succinate buffers were only 5 min in the 15 mM buffer and 9 min in the 5 mM buffer (Figure 6a). Similar results were obtained with caffeine release from HP-50-coated capsules (Figure 6b). The differences in the initial release time in 5 mM and 15 mM buffers were 7 min and 1 min, respectively. These results are in line with the previous findings and suggest that a succinate buffer at similar molarity to the bicarbonate buffer of interest can be chosen as surrogate buffer.

### 3.4. Dissolution of Diclofenac Sodium Tablets Coated with Methacrylic Acid/Ethyl Acrylate Copolymer (1:1)

**Figure 7:**
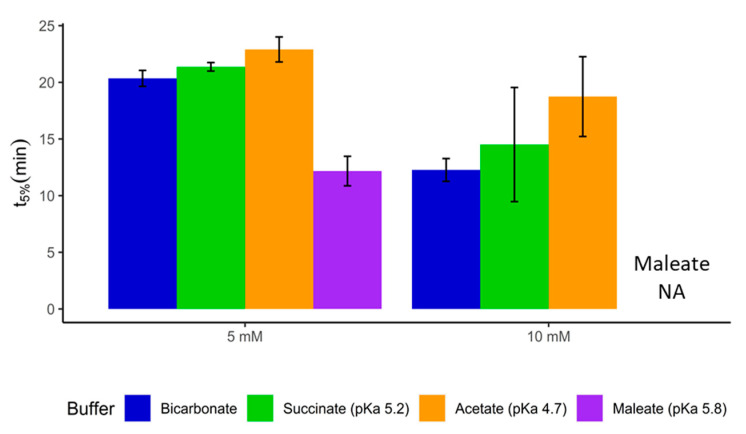
t_5%_ for diclofenac AbZ tablets in different buffers, all at pH 6.3. Buffer molarity is based on conjugate base. Release data in 10 mM maleate buffer are not available (NA).

Diclofenac AbZ tablets are coated with methacrylic acid/ethyl acrylate copolymer (1:1) and targets the proximal intestine. Buffer molarities were chosen according to jejunal bicarbonate concentrations reported in the literature [18,27], all at pH 6.3. As shown in Figure 7, the 5 mM maleate buffer overestimated the in vitro release time significantly (*p*-value < 0.001) in comparison to bicarbonate and was therefore not used as surrogate buffer in further testing. The dissolution in the 5 mM succinate media matched the release in bicarbonate fairly well, and the difference was not found to be significant (*p*-value 0.574). The t_5%_ value in 5 mM acetate buffer was shown to be significantly different from the bicarbonate buffer (*p*-value 0.042), although the absolute difference was only 2.6 min and therefore probably not physiologically relevant.

The 10 mM buffers showed a similar trend of dissolution. The rank order of t_5%_ values was again bicarbonate < succinate < acetate, although the differences were not significant (*p*-value > 0.05). These results are supportive of those in Section 3.1, wherein the pKa_eff_ of bicarbonate in the dissolution of EC formulations was dependent on the equilibrium between CO_2_ and HCO_3_^−^ in the boundary layer as well as in the gel layer, as well as the fact that succinate could be a valuable starting point for the development of biopredictive dissolution buffers for these products. This would also offer the opportunity to add, e.g., bile salts to the test media, which is not possible in a bicarbonate system because of foam forming [20]. This is of interest, especially for lipophilic drugs where the solubility of a drug could be affected by surfactants.

### 3.5. Dissolution of Claversal Tablets and Diclofenac AbZ Tablets with Increased Agitation Speed

**Figure 8:**
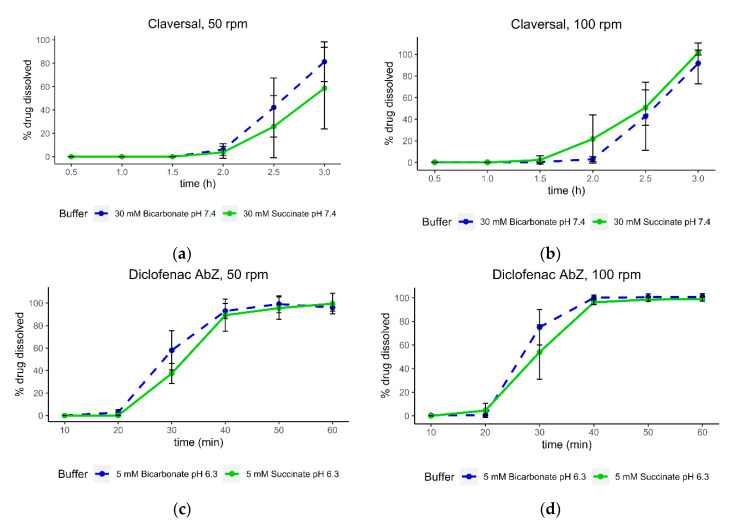
Dissolution of Claversal tablets in 30 mM bicarbonate and succinate buffer at pH 7.4 with (**a**) 50 rpm and (**b**) 100 rpm paddle speeds, and dissolution of diclofenac AbZ tablets in 5 mM bicarbonate and succinate buffer at pH 6.3 with (**c**) 50 rpm and (**d**) 100 rpm paddle speeds.

Dissolution experiments of Claversal and diclofenac AbZ tablets at 100 rpm were performed using bicarbonate and succinate buffers at 30 mM/pH 7.4 and 5 mM/pH 6.3, respectively. The dissolution data are depicted in Figure 8. The dissolution at 100 rpm was faster than at 50 rpm due to the higher agitation speed. At each agitation speed, the dissolution in succinate buffer matched that in bicarbonate buffer fairly well. Interestingly, the dissolution at 50 rpm slightly underpredicted the bicarbonate profile, while at 100 rpm a slightly faster dissolution was observed. Although the significance of this observation could not be confirmed due to the high variability, the effect of the paddle speed could be investigated in a further study, since it is in line with the effective pKa of bicarbonate in the diffusion layer, being expected to be lower at higher agitation speeds [9,28].

### 3.6. Establishing Surrogate Buffers

**Figure 9:**
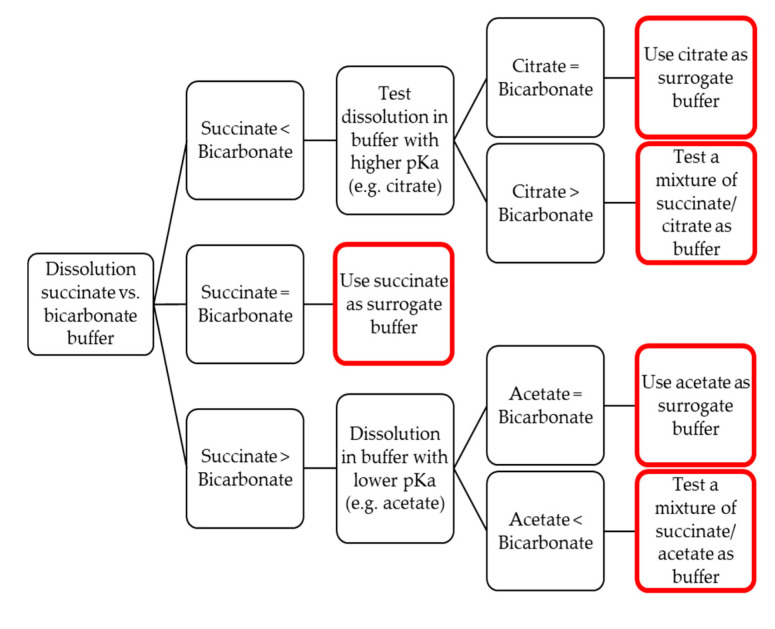
Graphical representation of establishing a surrogate buffer.

It seems that succinate constitutes an appropriate initial guess when developing a surrogate buffer for a relatively fast dissolving formulation, while citrate is a good starting point for a relatively slow dissolving one. This enables establishing a surrogate buffer algorithm (Figure 9) as follows. First, test the dissolution in bicarbonate and succinate buffer at similar conjugate base concentration and pH. In case the dissolution in the succinate buffer is slower (i.e., the pKa of succinate is lower than the effective interfacial pKa of bicarbonate), an experiment in, e.g., citrate buffer can be performed. If the dissolution in citrate buffer is too rapid, the interfacial pKa of bicarbonate is in between the two tested buffers and a mixture of succinate and citrate (at the same total conjugate base molarity) can be used as surrogate buffer. In the event that the dissolution in the succinate buffer is faster than in the bicarbonate buffer (i.e., the pKa of succinate is higher than the interfacial pKa of bicarbonate), the procedure similar to that described above, but instead of citrate, a buffer with a lower pKa (e.g., acetate) should be used. If the dissolution in acetate buffer is too slow, a mixture of acetate and succinate (at the same total buffer conjugate base concentration) can be used as surrogate buffer. For slower-dissolving Eudragit S-based formulations, citrate might be a better initial guess.

Such an algorithm will provide a systematic way for developing biopredictive media for dissolution testing of pH-dependent drug delivery systems with as few experimental iterations as possible. Expanding the database of tested products can provide a certain cutoff that will enable determining when to use succinate and when to use citrate as a starting buffer.

## 4. Conclusions

The dissolution of enteric polymers is, in contrast to small molecules, more complex due to chain disentanglement and gel layer development during dissolution. This is also the reason why the right choice of buffer has a significant influence on the dissolution of EC dosage forms, and it is important to account not only for the pH and buffer molarity but also the pKa of the buffer when designing media for dissolution of EC dosage forms. All in all, succinate and citrate buffers were shown to be viable as physiologically relevant candidate surrogate buffers for in vitro testing. Using these buffers as “initial guesses” enables a relatively simple development of biopredictive dissolution testing for pH-dependent drug delivery systems with minimal experimental iterations.

## Figures and Tables

**Table 1:** Average dose of paracetamol of the filled hydroxypropyl methylcellulose (HPMC) capsules and applied coating (based on dry polymer weight).

Batch	Dose	Coating Level
Eudragit S	220.7 ± 5.3 mg	5.50 mg/cm^2^
HPMCAS-LF	205.7 ± 2.8 mg	9.16 mg/cm^2^
HP-55	214.7 ± 3.0 mg	9.04 mg/cm^2^
HP-55S	214.7 ± 3.0 mg	6.92 mg/cm^2^
